# Decoding the power of the microbiome in human health

**DOI:** 10.3389/fcimb.2026.1877371

**Published:** 2026-08-21

**Authors:** Manoj Kumar, Noora Almohannadi, Souhaila Al Khodor

**Affiliations:** Research Department, Sidra Medicine, Doha, Qatar

**Keywords:** antimicrobial, chemotherapy modulation, clinical trial, functional microbial consortia, immune checkpoint inhibitors, immuno-modulator, microbial biomarkers, microbial dysbiosis

## Abstract

The human microbiota plays a vital role in maintaining physiological homeostasis and overall health. Microbial communities colonize distinct anatomical sites, including the gut, oral cavity, respiratory tract, and skin, where they engage in symbiotic interactions with the host. These site-specific microbial communities contribute to essential functions such as nutrient metabolism, vitamin and short-chain fatty acid (SCFA) synthesis, immune regulation, and epithelial barrier integrity. Disruption of this balance, known as dysbiosis, is increasingly linked to a wide range of diseases, including inflammatory bowel disease (IBD), obesity, diabetes, and cancer. In this review, we summarize the current understanding of the human microbiota, highlighting its role in vitamin biosynthesis, the gut–brain axis, and immune modulation. We further the role of microbiome alterations in disease pathogenesis and outline emerging microbiome-based therapeutic strategies aimed at restoring microbial homeostasis.

## Introduction

While the terms “microbiota” and “microbiome” are often used interchangeably, they have distinct definitions: “microbiota” refers to the community of microorganisms (bacteria, archaea, fungi, and viruses), whereas the “microbiome” encompasses their collective genomes (Berg et al., 2020; Kumar et al., 2020). Humans live in a symbiotic relationship with these microbes, which contribute to numerous vital physiological processes in exchange for habitat and nutrients (Ma et al., 2024). Each anatomical site harbors a unique microbial community shaped by factors such as pH, oxygen levels, moisture, and immune activity (Ma et al., 2024). Interestingly, microbial profiles tend to be more similar across individuals at the same body site than between different sites within the same individual (Gilbert et al., 2018).

The gut hosts the largest and most diverse microbial population, estimated at 100 trillion organisms (Qin et al., 2010). Often referred to as the “second human genome”, these microbes play critical roles in immune regulation, endocrine and neural signaling pathways (Grice and Segre, 2012). A healthy gut maintains a balance between beneficial and potentially pathogenic microbes, whereas disruption of this balance, termed microbial dysbiosis or imbalance, has been linked to various diseases (Kumar et al., 2021a; Sanchis-Artero et al., 2021; Hou et al., 2022; Chopra et al., 2024; Safarchi et al., 2025) such as inflammatory, metabolic and neurological disorders (Kumar et al., 2019; Elhag et al., 2022; Mikó et al., 2024). Emerging evidence suggests that these health effects are largely mediated by the microbial production of bioactive metabolites (Bishai and Palm, 2021). These compounds help regulate immune responses, reinforce epithelial barrier integrity, influence host metabolism, and facilitate inter-organ communication (Kumar et al., 2020). Given this complex interplay, microbiota-targeted strategies are increasingly being explored for disease prevention and therapeutic interventions (Gilbert et al., 2025). The following section examines key microbiota-derived strategies and their roles in health and disease modulation.

## The functional shift: from cataloging to metabolite production

Following the completion of the Human Genome Project in 2003 and the first complete human genome sequence in 2022, the release of the human pangenome has significantly enhanced our understanding of human genetic diversity (Aganezov et al., 2022; Hoyt et al., 2022; Nurk et al., 2022). In parallel, studies highlight the human microbiome, bacteria, fungi, archaea, viruses, and their genomes, as a critical partner in continuous crosstalk with the host genome (Group et al., 2009; Qin et al., 2010; Kumar et al., 2022). While human genetic variation explains only ~0.1% of inter-individual differences (Huang et al., 2015), the microbiome significantly shapes diverse phenotypes through dynamic interactions and the production of bioactive metabolites (Clemente et al., 2012; Hsu and Schnabl, 2023a). Advances following the Human Microbiome Project (HMP), have transitioned the field from primarily asking “who is there?” to exploring “what are they doing?” (Group et al., 2009). This shift emphasizes the functional properties of microbial communities. Computational analyses have uncovered over 14,000 biosynthetic gene clusters (BGCs) in the human microbiome (Cimermancic et al., 2014), highlighting an extensive potential to produce bioactive compounds, including enzymes and antimicrobial agents (Blin et al., 2013; Cimermancic et al., 2014; Donia et al., 2014; Donia and Fischbach, 2015). Further genome mining continues to uncover novel pathways, emphasizing the role of metabolites as primary mediators of host–microbe interactions (Cimermancic et al., 2014; Zheng et al., 2016).

The human microbiome produces a diverse array of metabolites and small molecules, including vitamins, short-chain fatty acids (SCFAs), neurotransmitters, lipopolysaccharides (LPS), and ribosomally synthesized and post-translationally modified peptides (RiPPs) (Cimermancic et al., 2014). Many of these compounds, including SCFAs and LPS, exist at pharmacologically relevant concentrations (Donia and Fischbach, 2015). The biochemical products of microbiota play key roles in modulating the immune system, metabolism, and neural functions, linking microbial activity to various health outcomes (Rooks and Garrett, 2016; Hou et al., 2022). For example, SCFAs such as butyrate are crucial for gut health, modulating inflammation and maintaining the integrity of the intestinal barrier (Du et al., 2024). Beyond microbial metabolites, host–microbiome communication is also mediated by microbial extracellular vesicles, which transport diverse bioactive molecules capable of modulating host immune and metabolic pathways (Toyofuku et al., 2023; Kumar et al., 2024). In addition, the gut microbiome signals through multiple organ-specific axes, including the gut–liver (Hsu and Schnabl, 2023b), gut–brain (Cryan et al., 2019; Jiang et al., 2026), and gut–lung axes (Cryan et al., 2019; Dang and Marsland, 2019; Liu et al., 2025), thereby influencing systemic physiology and disease pathogenesis. Collectively, these interconnected pathways underscore the multi-layered and systemic nature of host–microbiome interactions in health and disease. Moreover, microbial molecules can influence central nervous system function via the gut–brain axis, contributing to regulation of mood, behavior, and neuroimmune signaling (O’Riordan et al., 2025). Because metabolic changes occur rapidly and reflect the physiological state of both the host and its microbiota, these molecules serve as valuable biomarkers for diagnostics and offer mechanistic insights into disease associations (Gilbert et al., 2016). **Table 1** summarizes key metabolites and their potential roles in human health.

**Table 1:** Functional contributions of human gut microbes in vitamin and SCFA production.

Vitamin/compound	Key microbiota involved	Physiological role	Ref
Vitamin B1 (Thiamin)	*Prevotella spp., Desulfovibrio spp., Bacteroides spp.*	Coenzyme in carbohydrate metabolism, supports nervous system function	(Arumugam et al., 2011; Costliow and Degnan, 2017)
Vitamin B2 (Riboflavin)	*Bacteroidetes, Fusobacteria, Proteobacteria, Lactobacillus*	Cofactor in energy metabolism; antioxidant defense	(Magnúsdóttir et al., 2015; Thakur et al., 2016; García-Angulo, 2017)
Vitamin B5 (Pantothenic Acid)	*Escherichia coli, Salmonella typhimurium, Corynebacterium glutamicum*	Precursor of CoA; involved in TCA cycle, neuro-transmitter synthesis, and lipid metabolism	(Epelbaum et al., 1998, Leonardi and Jackowski, 2007; Hayflick, 2014)
Vitamin B6 (Pyridoxine)	*B. fragilis, Prevotella copri, Bifidobacterium longum, Collinsella aerofaciens, Helicobacter pylori*	Cofactor in amino acid metabolism and neurotransmitter synthesis	(Plecko and Stöckler, 2009; Arumugam et al., 2011; Yoshii et al., 2019)
Vitamin B7 (Biotin)	*B. fragilis, P. copri, Fusobacterium varium, Campylobacter coli, Bifidobacterium longum*	Coenzyme in fatty acid synthesis, amino acid metabolism	(Zempleni et al., 2009; Arumugam et al., 2011; Magnúsdóttir et al., 2015; Hayashi et al., 2017; Hossain et al., 2022)
Vitamin B9 (Folate)	*B. fragilis, P. copri, Clostridium difficile, Lactobacillus spp., Bifidobacterium spp., Fusobacterium varium, Salmonella enterica*	DNA synthesis, cell division, methylation reactions	(Magnúsdóttir et al., 2015; Mahmoud and Ali, 2019; Hossain et al., 2022)
Vitamin B12 (Cobalamin)	*Pseudomonas denitrificans, Bacillus megateriu, Propionibacterium freudenreichii, Bacteroides, Clostridium, Faecalibacterium, Bifidobacterium*	Red blood cell formation, nervous system function, coenzyme in metabolic reactions	(Gu et al., 2015; Song et al., 2016; Piwowarek et al., 2018; Kelly et al., 2019; Hossain et al., 2022)
Vitamin K	*Bacteroides ovatus, Prevotella buccae, E. coli, Enterococcus faecalis, Staphylococcus spp., Citrobacter freundii*	Blood clotting, bone health, potential cell signaling	(Cooke et al., 2006; Akbari and Rasouli-Ghahroudi, 2018; Ellis et al., 2021; Mladěnka et al., 2022)
SCFAs	Acetate: *Blautia hydrogenotrophica* Propionate: *Akkermansia muciniphila* Butyrate: *Faecalibacterium prausnitzii, Eubacterium rectale, Eubacterium hallii, and Ruminococcus bromii*	Immune regulation, gut barrier integrity, metabolic health, Energy source, barrier protection, anti-inflammatory effects	(Pryde et al., 2002; Louis et al., 2010; Louis et al., 2014; Thursby and Juge, 2017; Liu et al., 2022)

## Microbiota-derived vitamins and short-chain fatty acids

**Figure 1:**
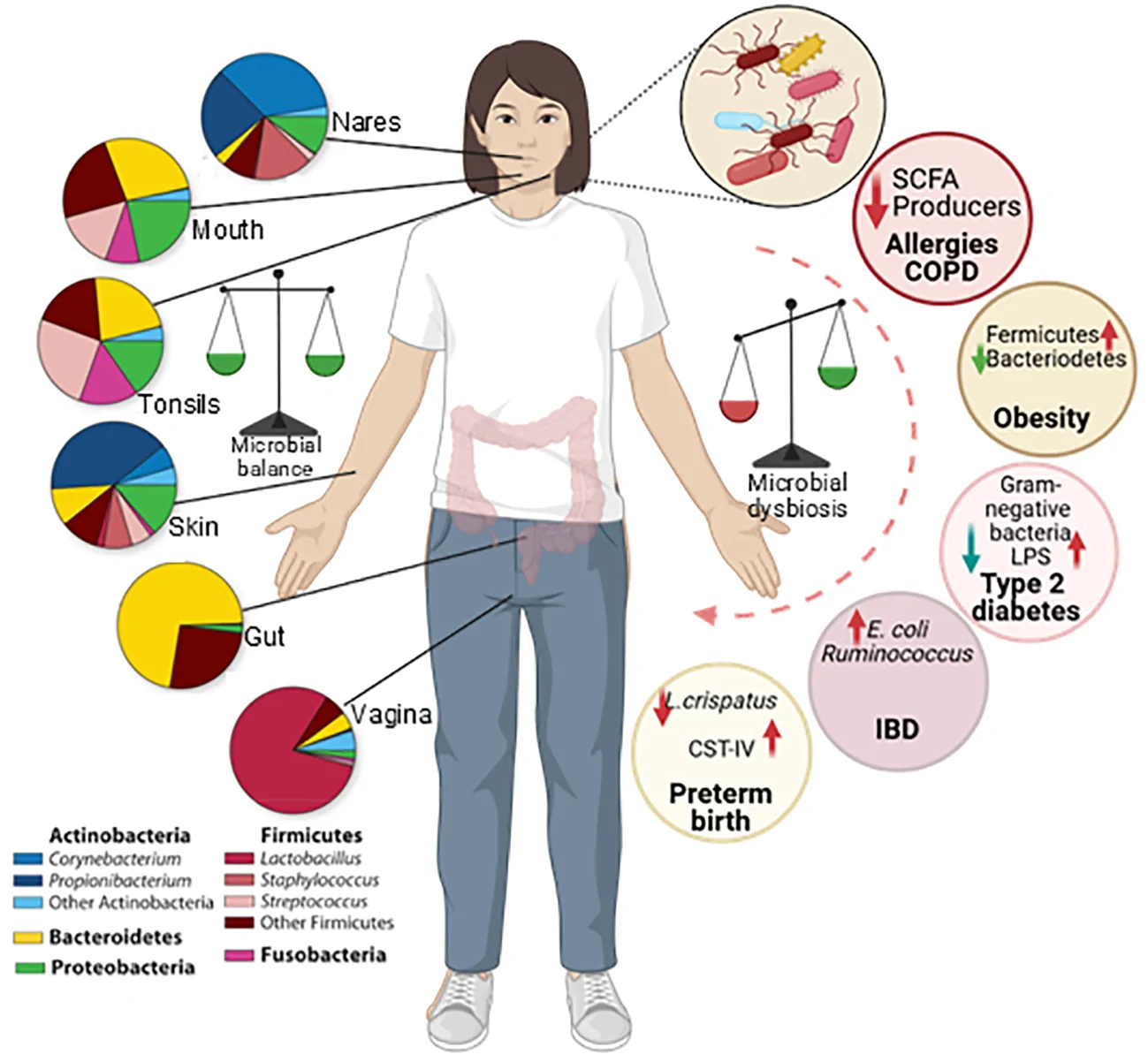
Microbiome composition at different body sites and their role in disease. Distinct microbial communities inhabit different body sites, maintaining local homeostasis. Dysbiosis at these sites can disrupt host functions and contribute to diseases such as IBD, obesity, and diabetes.

Each human body site harbors a distinct and dynamic microbial community essential for homeostasis, with composition varying based on local environmental factors (**Figure 1**). In the gut these microbes perform essential functions, including fermenting dietary fibers, protecting against pathogens, regulating immune responses, and producing key metabolites such as SCFAs that support gut barrier integrity and modulate inflammation (Tan et al., 2014; Xiong et al., 2022). In addition, specific microbial species are involved in the biosynthesis of B-complex vitamins, including thiamin (B1) (Arumugam et al., 2011), riboflavin (B2) (Magnúsdóttir et al., 2015; Thakur et al., 2016; García-Angulo, 2017), pantothenic acid (B5) (Epelbaum et al., 1998), pyridoxine (B6) (Yoshii et al., 2019), biotin (B7) (Magnúsdóttir et al., 2015), folate (B9) (Magnúsdóttir et al., 2015; Hossain et al., 2022), and cobalamin (B12) among others (Gu et al., 2015; Song et al., 2016; Piwowarek et al., 2018). These vitamins are essential for numerous physiological processes such as enzymatic activity, DNA synthesis, neural development, and immune function (**Table 1**). For instance, *Prevotella* and *Bacteroides* are involved in thiamin and folate production (Magnúsdóttir et al., 2015; Hossain et al., 2022), while *Escherichia coli* and *Salmonella typhimurium* synthesize pantothenic acid (Epelbaum et al., 1998). Riboflavin is produced by a broad spectrum of gut and lactic acid bacteria (Magnúsdóttir et al., 2015; Thakur et al., 2016; García-Angulo, 2017). Notably, while only 20% of gut microbes can synthesize vitamin B12, this nutrient supports the metabolism of over 80% of the surrounding microbial community, illustrating a complex intra-microbiome dependency. Furthermore, vitamin K—essential for coagulation—is synthesized by bacteria such as *Bacteroides ovatus*, *E. coli*, and *Staphylococcus* spp (Cooke et al., 2006; Ellis et al., 2021).

Beyond vitamin biosynthesis, gut microbes ferment dietary fibers to produce SCFAs like acetate, propionate, and butyrate (Pryde et al., 2002). These metabolites, generated predominantly by *Faecalibacterium prausnitzii* (Louis et al., 2010; Thursby and Juge, 2017), *Akkermansia muciniphila* (Louis et al., 2014; Liu et al., 2022), and *Blautia hydrogenotrophica*, exert extensive benefits, including anti-inflammatory, immunoregulatory, neuroprotective, and metabolic effects. SCFAs also reinforce gut barrier integrity and modulate host-microbe interactions critical for immune homeostasis (Tan et al., 2014; Xiong et al., 2022). Disruptions in the gut microbial community can impair the synthesis of these compounds, contributing to inflammatory bowel disease (IBD), metabolic syndrome, and neurodevelopmental conditions (Belcheva et al., 2014; Kumar et al., 2019; Singh et al., 2019; Elhag et al., 2021).

## Microbial metabolites as mediators of host physiology and health

Beyond vitamins and SCFAs, microbiota generate a diverse range of bioactive compounds, including antimicrobial agents, immunomodulators, and neuroactive molecules (Kumar et al., 2020). Commensal bacteria produce a variety of lantibiotics, such as ruminococcin A, microcin, lactocillin, and epidermin, that suppress pathogens and help maintain microbial balance across the gut, skin, and vaginal mucosa (Donia et al., 2014; Chopra et al., 2022; Shah et al., 2025). Other components, such as LPS and glycolipids, interact directly with host immune cells to influence immune responses. **Table 2** summarizes key metabolites produced by microbiota species, highlighting their roles in both microbe-microbe and host-microbe interactions.

**Table 2:** Microbiota-derived metabolites and their biological functions.

Type	Metabolite	Producer microbe(s)	Primary location	Function(s)
Antimicrobial	Ruminococcin A	*Ruminococcus gnavus*	Gut	Bacteriocin with Gram-positive activity
	Microcin	*Escherichia coli*	Gut	Antimicrobial peptide targeting enteric pathogens
	Clostridiolysin S	*Clostridium sporogenes*	Gut	Cytolytic against Gram-positive bacteria
	Zwittermicin	*Bacillus cereus*	Gut	Broad-spectrum antimicrobial; produced under specific conditions
	Cytolysin	*Enterococcus faecalis*	Gut	Lytic to other bacteria and eukaryotic cells
	Lactocillin	*Lactobacillus gasseri*	Vagina	Novel antibiotic peptide; Inhibits vaginal pathogens
	Epidermin	*Staphylococcus epidermidis*	Skin	Bacteriocin against skin pathogens
	SA-FF22	*Streptococcus pyogenes*	Skin/Oral	Bacteriocin with broad-spectrum activity
	Streptolysin	*Streptococcus pyogenes*	Skin/Oral	Cytotoxic to immune cells
Immuno-modulatory	Indolepropionic acid	*Clostridium sporogenes*	Gut	Antioxidant and neuroprotective; gut-brain signaling
	Polysaccharide A	*Bacteroides fragilis*	Gut	Induces Treg cells; anti-inflammatory
	α-galacto-sylceramide	*Bacteroides fragilis*	Gut	Activates NKT cells; modulates immunity
	Cereulide	*Bacillus cereus*	Gut	Mitochondrial toxin; can induce vomiting
Lipids	LPS, peptidoglycan, acylglycerols, triglycerides	*E. coli, Bifidobacterium, Roseburia, Lactobacillus, Clostridium spp.*	Gut	Immune signaling; modulation of innate immunity
Polyamines	Putrescine, Cadaverine	*Campylobacter jejuni, Clostridium saccharolyticum*	Gut	Affect gut physiology and host immune response
Peptides	Muramyl di-/tripeptides	*Fusobacterium nucleatum*	Oral	Immune activation; associated with inflammation
Mycolic Lipids	Mycolic acid	*Mycobacterium spp.*	Lung	Immune modulation; granuloma formation
	Mycolactone	*Mycobacterium ulcerans*	Skin	Cytotoxic and immunosuppressive
	Staphyloxanthin	*Staphylococcus aureus*	Skin	Antioxidant; protects against host immune attack
Neuro-transmitters	γ-Aminobutyric acid (GABA)	*Lactobacillus, Bifidobacterium spp.*	Gut	Inhibitory neurotransmitter; reduces anxiety, gut motility modulation
	Serotonin (5-HT, indirectly)	*Enterococcus, Streptococcus spp.*	Gut	Regulates mood, gut motility, and secretion
	Dopamine	*Escherichia coli, Bacillus spp.*	Gut	Modulates behavior, motivation, and movement
	Norepinephrine	*Escherichia coli*	Gut	Influences gut motility, host stress response
	Acetylcholine	*Lactobacillus spp.*	Gut	Modulates muscle contraction, inflammation, and cognition

Specific species produce immunomodulatory molecules, such as polysaccharide A, α-galactosylceramide, and indolepropionic acid, which promote regulatory T cell development and maintain intestinal homeostasis (Wieland Brown et al., 2013; Zheng et al., 2020; Schütz et al., 2025; Zhao et al., 2025). Other lipid-based molecules such as LPS and peptidoglycan fragments interact with host pattern recognition receptors to modulate inflammation and barrier function (Li and Wu, 2021), while polyamines and peptide derivatives support epithelial renewal and immune activation (Zheng et al., 2020). Importantly, emerging evidence highlights that several gut microbes can synthesize or modulate neurotransmitters such as gamma-aminobutyric acid (GABA), serotonin, dopamine, and acetylcholine—chemical messengers that influence behavior, mood, cognition, and gut–brain communication through direct and indirect pathways (Ashique et al., 2024). Disruptions to these metabolite pathways, caused by microbial imbalance, antibiotics exposure, or other perturbations, can impair host physiology and contribute to a wide range of conditions, including infections, chronic inflammation, metabolic disorders, and neuropsychiatric diseases (Loh et al., 2024).

In addition to these endogenous functions, environmental microbes, especially those in soil and marine ecosystems, play a crucial indirect benefit to human health as a source of antibiotics. Members of the genus Streptomyces (e.g., *S. griseus*, *S. aureofaciens*, *S. erythraeus*, *S. orientalis*, and *S. rifamycinica*) have given rise to several life-saving antibiotics, including streptomycin (Schatz et al., 1944), tetracycline (Duggar, 1948), erythromycin (Oliynyk et al., 2007), vancomycin (McCormick et al., 1955), and rifampicin (Sensi, 1983). Other important antibiotic producers include *Micromonospora purpurea* (gentamicin) (Gonzalez et al., 1995), *Bacillus subtilis* (bacitracin) (Rietkötter et al., 2008), *B. polymyxa* (polymyxins) (Shaheen et al., 2011), and the fungus *Penicillium notatun* and *Penicillium chrysogenum*, which produces penicillin (Shaheen et al., 2011) (**Supplementary Table 1**). These compounds evolved as competitive survival strategies in nutrient-limited environments and remain foundational to modern medicine (Cycoń et al., 2019).

## Microbial-immune interactions in health and disease

The interaction between the immune system and the microbiota begins at birth—potentially even earlier through prenatal microbial exposure and persists throughout life (Kumar et al., 2021b; Kumar et al., 2022; Saadaoui et al., 2024). Intestinal epithelial cells (IECs) serve as the primary interface, equipped with pattern recognition receptors (PRRs) such as Toll-like receptors (TLRs) and NOD2, which detect microbial signals and initiate immune responses (Akira et al., 2001; McDermott and Huffnagle, 2014; Soderholm and Pedicord, 2019). IECs secrete antimicrobial peptides (AMPs), cytokines, chemokines, and hormones that enhance barrier function and pathogen clearance (Thaiss et al., 2016). Microbial signals, originating from live bacteria, dead cells, or metabolites, activate intracellular pathways that are essential for mucosal immunity and barrier integrity (Parker et al., 2020).

Microbial imbalance is increasingly associated with chronic immune-mediated and metabolic disorders such as IBD, celiac disease, multiple sclerosis, rheumatoid arthritis, asthma, diabetes, and colorectal cancer (CRC) (Al Khodor and Shatat, 2017; Baohong Wang et al., 2017; Levy et al., 2017; Augustine et al., 2023). In CRC, the microbiome is often enriched with pathogenic taxa like *Pseudomonas* and *Helicobacter*, while beneficial butyrate producers are depleted (Zackular et al., 2013). During advanced stages, species such as *Bacteroides massiliensis* and *E. coli* exacerbate inflammation and promote disease progression through microbial translocation (Kostic et al., 2013; Rubinstein et al., 2013; Feng et al., 2015). Similar microbial imbalances occur during infections, where pathogen overgrowth disrupts the epithelial barrier and drives excessive inflammation (Brenchley and Douek, 2012; Hand et al., 2012; Kumar et al., 2019). **Table 3** summarizes the role of various microbial species in human health and disease, emphasizing their dual contributions to homeostasis and pathology.

**Table 3:** Microbial species associated with human diseases and their potential role in human health.

Microbe (genus/species)	Primary location	Associated disease(s)	Role in human health	Proposed mechanism	Ref
*Corynebacterium spp.*	Skin, Nasal	Increased in atopic dermatitis	Modulates skin immune responses; can contribute to barrier dysfunction and inflammation	Proteases/lipases trigger Th2 inflammation and barrier stress	(Bjerre et al., 2021; Ottria et al., 2026)
*Bifidobacterium longum*	Gut	Decreased in atopic diseases; IBD	Supports gut-brain communication; ferments oligosaccharides; produces vitamins and SCFAs	Acetate/lactate support barrier; promotes Treg/IL-10 tone	(Loh et al., 2024; Seong et al., 2025)
*Streptococcus salivarius*	Oral	Decreased in Oral dysbiosis; halitosis	Produces bacteriocins; promotes colonization resistance; used in oral probiotics	Bacteriocins + competition suppress oral pathobionts	(Karbalaei et al., 2021; Ottria et al., 2026)
*Eggerthella lenta*	Gut	Increased in bacteremia; IBD	Metabolizes host compounds and drugs; modulates immune responses	Drug metabolism reshapes host metabolites and immune tone	(Ganamurali and Sabarathinam, 2025; Shin et al., 2025)
*Ureaplasma parvum*	Genital tract	Increased in pregnancy such as Chorioamnionitis; preterm birth	Stimulates cytokine and prostaglandin production; linked to premature labor and membrane rupture	Innate activation → ↑ cytokines/prostaglandins driving PTB pathways	(Sprong et al., 2020; Kumar et al., 2021a)
*Chlamydia trachomatis*	Genital tract	Increased in pregnancy complications; cervical cancer	Induces chronic inflammation; disrupts epithelial and placental barriers	Persistent infection → chronic inflammation and epithelial injury	(Zhu et al., 2016; Kumar et al., 2021a; Ottria et al., 2026)
*Lactobacillus jensenii*	Genital tract	Decreased in bacterial vaginosis	Produces lactic acid and H_2_O_2_; maintains vaginal pH and inhibits pathogens	Lactic acid/H_2_O_2_ maintain low pH; inhibit BV pathogens	(Tachedjian et al., 2017; Amabebe and Anumba, 2018; Ottria et al., 2026)
*Turicibacter sanguinis*	Gut	Decreased in depression; anxiety	Produces SCFAs; modulates serotonin pathways; supports gut-brain axis	SCFAs influence enteroendocrine signaling and serotonin pathways	(Margolis et al., 2021; Loh et al., 2024)
*Clostridium scindens*	Gut	Decreased in dysbiosis-related infections; C. difficile	Converts bile acids; promotes colonization resistance	Secondary bile acids inhibit C. difficile; modulate FXR/TGR5	(Saenz et al., 2023)
*Salmonella enterica*	Gut	Increased in gallbladder cancer	Chronic inflammation; associated with carcinogenesis via DNA damage	Chronic carriage → inflammation/ROS, pro-tumor microenvironment	(Koshiol et al., 2016; Ottria et al., 2026)
*Lactobacillus crispatus*	Genital tract	Decreased in Bacterial vaginosis; preterm birth	Produces lactic acid; maintains vaginal microbiome stability	D-lactic acid lowers pH; stabilizes mucosa, limits BV	(Amabebe and Anumba, 2018)
*Lactobacillus rhamnosus*	Gut, Genital tract, Oral	Decreased in GI infections; urogenital infections	Common probiotic; inhibits pathogens; enhances host immunity	Strengthens barrier; modulates DC/Treg/IgA responses	(Amabebe and Anumba, 2018; Liu et al., 2023)
*Anaerostipes spp.*	Gut	Decreased in metabolic syndrome	Converts lactate to butyrate; promotes epithelial health	Lactate→butyrate cross-feeding; improves barrier and inflammation	(Kumar et al., 2019; Bui et al., 2021)
*Blautia spp.*	Gut	Decreased in metabolic syndrome	Produces SCFAs; associated with anti-inflammatory profiles	SCFAs support anti-inflammatory cytokine and metabolic balance	(Kumar et al., 2019; Liu et al., 2021)
*Oscillospira spp.*	Gut	Decreased in obesity	Linked to metabolic leanness and reduced inflammation	Fiber fermentation linked to lower endotoxemia/inflammation	(Chen et al., 2020)
*Parabacteroides distasonis*	Gut	Decreased in obesity; Inflammatory diseases	Produces acetate; improves metabolic and immune balance	SCFAs + bile-acid modulation improves immune-metabolic tone	(Sun et al., 2023)
*Akkermansia muciniphila*	Gut	Decreased in obesity; Type 2 Diabetes	Degrades mucin; strengthens gut barrier; improves metabolic health	Mucin use supports mucus layer; improves tight junctions	(Lakshmanan et al., 2022)
*Gardnerella vaginalis*	Genital tract	Increased in bacterial vaginosis	Disrupts vaginal epithelium; increases susceptibility to STIs, miscarriage, and preterm birth	Biofilm + vaginolysin disrupt epithelium; raise pH/inflammation	(Kumar et al., 2021a; Anton et al., 2022)
*Alistipes spp.*	Gut	Increased in CDC; depression	May modulate immune signaling; impacts gut–brain axis	Alters bile/amino-acid metabolites affecting HPA/cytokines	(Parker et al., 2020)
*Bacteroides ovatus*	Gut	Decreased in CRC	Produces anti-inflammatory polysaccharides; supports mucosal health	Polysaccharides promote mucosal tolerance and homeostasis	(Feng et al., 2015)
*Bacteroides massiliensis*	Gut	Increased in CRC advanced	Associated with tumor progression; potential pro-inflammatory mediator	CRC enrichment associated with pro-inflammatory ecology	(Feng et al., 2015; Xie et al., 2024)
*Bacteroides fragilis (toxigenic)*	Gut	Increased in CRC; CD	Secretes BFT toxin; induces epithelial proliferation and inflammation	BFT cleaves E-cadherin → IL-17 inflammation/tumor signals	(Kitamoto et al., 2020; Giampazolias et al., 2024)
*Acinetobacter spp.*	Gut, skin	Increased in CRC; hospital-acquired infections	Linked to dysbiosis; can exacerbate inflammation	LPS-driven innate activation amplifies dysbiosis inflammation	(Soranno et al., 2025)
*Bacteroides vulgatus*	Gut	Decreased in CRC; IBD	Produces pro-inflammatory lipopolysaccharides; linked to disease progression	Pro-inflammatory LPS promotes mucosal immune activation	(Wang et al., 2022)
*Pseudomonas aeruginosa mannose-sensitive hemagglutinin*	Gut, lungs	CRC; infections	Enhances anti-PD-1 efficacy via activation of tumor-infiltrating CD8^+^ T cells	DC activation → ↑ CD8^+^ priming and IFN-γ (ICI)	(Choi et al., 2023; Chen et al., 2025)
Fusobacterium nucleatum**	Gut	Increased in CRC	Biomarker of cancer risk; promotes immune evasion and tumor growth	FadA adhesion + Fap2 immune inhibition (NK/T cells)	(Kostic et al., 2013; Rubinstein et al., 2013; Mima et al., 2015; Piccinno et al., 2025)
*Parvimonas micra*	Gut	Increased in CRC	Biomarker of cancer risk; implicated in tumor microenvironment modulation	Enriches inflammatory niches; supports CRC-associated consortia	(Piccinno et al., 2025)
*Gemella morbillorum*	Gut	Increased in CRC	Biomarker of cancer risk/progression	Opportunistic enrichment linked to mucosal inflammation	(Piccinno et al., 2025)
*Peptostreptococcus stomatis*	Gut	Increased in CRC	Biomarker of cancer risk; linked to inflammatory responses	Innate stimulation in CRC niches; supports dysbiotic consortia	(Piccinno et al., 2025)
*Hungatella hathewayi*	Gut	Increased in CRC	Biomarker of cancer risk; possibly contributes to mucosal inflammation	Dysbiosis marker; inflammatory metabolite patterns association	(Piccinno et al., 2025)
*Ruminococcus bromii*	Gut	Increased in CRC	Biomarker of cancer risk/progression	Starch degradation reshapes metabolism in CRC dysbiosis	(Roelands et al., 2023)
Escherichia coli (pks+ strains)*	Gut	Increased in CRC; IBD	Produces genotoxin colibactin; induces DNA damage and inflammation	Colibactin genotoxicity → DNA damage + inflammation	(Jans and Vereecke; Palmela et al., 2018)
*Dorea spp.*	Gut	Increased in mental health disorders	Produces fermentation byproducts; influences gut-brain signaling	Fermentation byproducts alter gut–brain and inflammatory signaling	(Grimaldi et al., 2018)
*Phascolarctobacterium spp.*	Gut	Increased in mental health; metabolic diseases	Produces propionate; modulates gut–brain axis and host metabolism	Propionate signals via GPCRs; metabolic/neuro effects	(Xiong et al., 2023)
*Faecalibacterium prausnitzii*	Gut	Decreased in CRC; IBD; metabolic disorders	Major butyrate producer; strong anti-inflammatory effects	Butyrate + anti-inflammatory metabolites suppress NF-κB	(Fujimoto et al., 2013; Qin et al., 2025)
*Eubacterium rectale*	Gut	Decreased in IBD; metabolic syndrome	Produces butyrate; supports colonocyte health	Butyrate supports colonocytes; reduces mucosal inflammation	(Ning et al., 2023)
*Helicobacter pylori*	Gut	Increased in gastric cancer; MALT lymphoma	Biomarker of cancer risk/progression, Induces chronic inflammation via CagA and VacA toxins	CagA/VacA drive chronic inflammation and epithelial dysfunction	(IARC Working Group on the Evaluation of Carcinogenic Risks to Humans, 1994; Duan et al., 2025)
*Proteus mirabilis*	Gut	Increased in IBD	Disrupts epithelial integrity; promotes inflammatory responses	Motility/urease/LPS disrupt barrier; amplify innate inflammation	(Zhang et al., 2021)
*Actinomyces sp. oral taxon 181*	Gut	Increased in IBD	Oral pathobiont; translocates and triggers gut inflammation	Oral-to-gut translocation triggers Th17/innate responses	(Zheng et al., 2024; Li et al., 2026)
*Collinsella aerofaciens*	Gut	Increased in cardiovascular disease; IBD	Linked to elevated pro-inflammatory cytokines; impacts lipid metabolism	Bile/lipid shifts associate with IL-6/IL-17 signatures	(Wang et al., 2022)
*Desulfovibrio spp.*	Gut	Increased in IBD; UC	Sulfate reducer; produces H_2_S, damaging epithelial cells and promoting inflammation	H_2_S damages mucus/epithelium; promotes inflammation	(Xie et al., 2024)
*B. hansenii*	Gut	Increased in UC	Involved in mucin degradation; may impair mucus barrier	Mucin degradation thins mucus barrier; increases antigen exposure	(Zheng et al., 2024)
*Clostridium spiroforme*	Gut	Increased in UC	Toxin producer; disrupts immune homeostasis and epithelial integrity	Toxin-mediated epithelial injury promotes inflammation/leakiness	(Zheng et al., 2024)
*Gemella morbillorum*	Gut	Increased in UC	Opportunistic pathogen; associated with mucosal inflammation	Opportunistic overgrowth amplifies innate immune activation	(Zhang et al., 2021)
*Bacteroides obeum*	Gut	Decreased in CD	Modulates bile acid metabolism; may protect against inflammation	Bile-acid transformations support anti-inflammatory metabolites	(Zheng et al., 2024; Islam et al., 2026)
*Dorea formicigenerans*	Gut	Decreased in CD	Carbohydrate fermenter; supports SCFA-mediated epithelial health	Fermentation supports SCFA supply and epithelial energy	(Zheng et al., 2024)
*Eubacterium sp. CAG: 274*	Gut	Decreased in CD	Produces SCFAs; contributes to gut barrier function	SCFAs support barrier repair and immune regulation	(Mukherjee et al., 2020)
*Lawsonibacter asaccharolyticus*	Gut	Decreased in CD	Butyrate producer; supports epithelial integrity	Butyrate supports barrier integrity and Treg tone	(Zheng et al., 2024)
*Roseburia intestinalis*	Gut	Decreased in CD	Butyrate producer; associated with reduced gut inflammation	Butyrate supports mucus and tight junctions	(Nie et al., 2021)
*Bacteroides thetaiotaomicro*n	Gut	Decreased in IBD	Digests complex polysaccharides; promotes regulatory immune responses	Polysaccharide metabolism supports tolerance and homeostasis	(Schaus et al., 2024)
*Clostridium leptum*	Gut	Decreased in IBD	SCFA producer; induces regulatory T cells	SCFAs promote Treg differentiation; dampen inflammation	(Zheng et al., 2024)
*Roseburia inulinivorans*	Gut	Decreased in IBD	Butyrate producer; supports mucosal health	Inulin→butyrate supports mucosal repair	(Takahashi et al., 2016)
*Lachnospira spp.*	Gut	Decreased in IBD; allergy	Produces SCFAs; enhances immune regulation	SCFAs strengthen barrier and promote immune regulation	(De Filippis et al., 2021)
*Clostridium cluster IV/XIVa*	Gut	Decreased in IBD; dysbiosis	Induces regulatory T cells; major SCFA producers	Major butyrate producers; induce Foxp3^+^ Tregs	(Van den Abbeele et al., 2013)
*Ruminococcus torques, Ruminococcus gnavus*	Gut	Decreased in IBD; metabolic disorders	Fiber degraders; support SCFA production and gut barrier integrity	Glycan use reshapes mucus/SCFA balance (context-dependent)	(Lloyd-Price et al., 2019)
*Veillonella spp.*	Oral, Gut	Decreased in IBD; Oral disease	Converts lactate to propionate; helps maintain pH balance	Lactate→propionate cross-feeding stabilizes pH/metabolites	(Kumar et al., 2019)
*Ruminococcus torques*	Gut	Decreased in UC	Associated with impaired barrier function	Mucin/glycan shifts associated with barrier dysfunction	(Schaus et al., 2024)
*Bilophila wadsworthia*	Gut	Decreased in UC	Sulfite reducer; linked to inflammation	Sulfur metabolism promotes inflammatory mucosal responses	(Zheng et al., 2024)
*Faecalibacterium saccharivorans*	Gut	Decreased in UC	Butyrate producer; anti-inflammatory	Butyrate protects epithelium; supports anti-inflammatory tone	(Zheng et al., 2024)
*Gordonibacter formicilis*	Gut	Decreased in UC	Metabolizes polyphenols; potential immune modulator	Polyphenol metabolites may modulate immune signaling	(Zheng et al., 2024)
*Odoribacter splanchnicus*	Gut	Decreased in UC	Produces butyrate; supports mucosal integrity	Butyrate supports mucosal integrity and repair	(Zheng et al., 2024)
*Prevotella spp.*	Oral, Gut	Increased in periodontal disease; IBD	Ferments complex carbohydrates; interact with immune cells	Enrichment linked to Th17-skewed inflammation (context-dependent)	(Piccinno et al., 2025)
*B. fragilis, A. muciniphila, Bifidobacterium spp.*	Gut	High abundance modulates ICI responsiveness	Enhance ICI efficacy; reverse resistance; mitigate colitis	↑ antigen presentation/CD8^+^ priming; barrier support reduces colitis	(Li et al., 2024; Liu et al., 2025)
EDP1503	Gut	High abundance modulates ICI responsiveness	Enhance ICI efficacy; reverse resistance; mitigate colitis	Enhance tumor immune activation while limiting gut inflammation	NCT03775850 (Lei et al., 2025)
MET-4	Gut	High abundance modulates ICI responsiveness	Enhance ICI efficacy; reverse resistance; mitigate colitis	Restores taxa/metabolites; supports DC/CD8^+^ activation + barrier	(Spreafico et al., 2023)
VE800	Gut	High abundance modulates ICI therapy response	Enhance ICI efficacy; reverse resistance; mitigate colitis	Promotes inflamed TME; protects gut barrier to reduce toxicity	(Lyon De Ana et al., 2024; Wong et al., 2025)
*Faecalibacterium, Firmicutes*	Gut	High abundance and specific taxa modulate anti-CTLA-4 therapy response	Enhance ICI efficacy; Correlate with therapeutic efficacy and reduced toxicity	SCFA networks support response and lower toxicity	(Chaput et al., 2017)
*A. muciniphila, E. hirae, F. prausnitzii, B. longum, C. aerofaciens, E. faecium*	Gut	High abundance and specific taxa modulate anti-PD-1/PD-L1 response	Enhance ICI efficacy and survival	DC activation + CD8^+^ infiltration; SCFAs/mucus support response	(Derosa et al., 2018; Routy et al., 2018; Chen Y. et al., 2026)

IBD, Inflammatory Bowel Disease; UC, Ulcerative Colitis; ICI, Immune Checkpoint Inhibitor; CTLA-4, Cytotoxic T-Lymphocyte-Associated Antigen 4; PD-1/PD-L1, Programmed Cell Death-1/Programmed Death-Ligand 1; CRC, Colorectal cancer.

*Escherichia coli (pks^+^ strains) refers to specific strains of *E. coli* that carry the “pks” genomic island, a cluster of genes responsible for the biosynthesis of a genotoxin called colibactin. This strain is associated with an increased risk of colorectal cancer.

**Multiple strains of *F. nucleatum* have been linked to CRC risk such as *F. nucleatum* SGBs (SGB6007 (Fna C2), *F. nucleatum* animalis, SGB6014 *F. nucleatum* vincentii, SGB6011 *F. nucleatum *subsp*. nucleatum* (*F. nucleatum sensu stricto*), SGB6001 *F. nucleatum polymorphum*, SGB6013 (Fna C1) *F. nucleatum vincentii.*

## Microbiota–gut–brain interactions in health and disease

Despite their anatomical separation, the gut and the central nervous system (CNS) maintain a bidirectional communication network known as the gut–brain axis (Loh et al., 2024). The gut microbiota and its metabolites represent a key upstream regulator of CNS function, acting through metabolic, immune, and neural pathways (Morais et al., 2021; Doenyas et al., 2025; Ohara and Hsiao, 2025).

Microbiota-derived metabolites, including SCFAs, tryptophan derivatives, and secondary bile acids, can enter systemic circulation and influence brain function either directly or indirectly. These metabolites may cross the blood–brain barrier or signal through peripheral immune and neural pathways to regulate microglial maturation, neuroinflammation, neurotransmitter synthesis, and neuronal activity (Agus et al., 2018; Dalile et al., 2019; Loh et al., 2024). In particular, SCFAs such as butyrate exert neuroactive effects through histone deacetylase (HDAC) inhibition, promotion of regulatory T-cell differentiation, and strengthening of epithelial barrier integrity, thereby reducing systemic inflammation and indirectly supporting CNS homeostasis (Silva et al., 2020). Similarly, microbial metabolism of tryptophan generates indole derivatives that activate the aryl hydrocarbon receptor (AhR), influencing neuroimmune signaling and serotonin balance (Lamas et al., 2018; Gao et al., 2020). Dysbiosis may further exacerbate CNS dysfunction by increasing intestinal permeability and facilitating translocation of microbial products such as LPS, which activates NF-κB–mediated inflammatory pathways and contributes to microglial activation (Park et al., 2025; Sie and Tropini, 2026).

Conversely, the CNS exerts top-down regulatory control over gut physiology and microbial composition through neural, endocrine, and immune pathways (Morais et al., 2021; Doenyas et al., 2025; Ohara and Hsiao, 2025). The autonomic nervous system and hypothalamic–pituitary–adrenal (HPA) axis plays central roles in modulating intestinal function, including epithelial barrier integrity, immune activity, and gut motility (Rusch et al., 2023; Bertollo et al., 2025; Park et al., 2025). Stress-induced neuroendocrine signaling can alter microbial composition and promote dysbiosis, thereby reinforcing bidirectional gut–brain communication (Sie and Tropini, 2026). Neural inputs, including vagal and spinal afferents, regulate intestinal secretion and motility, while nociceptor signaling can stimulate goblet cell–mediated mucus secretion to modulate the mucosal barrier (Silva et al., 2020). In addition, vagal signaling influences enterochromaffin cell activity and serotonin release, which in turn affects gut microbial dynamics and intestinal physiology (Lamas et al., 2018; Gao et al., 2020).

Early experimental evidence by Sudo et al. (Sudo et al., 2004) demonstrated that germ-free mice exhibit exaggerated HPA axis responses to stress, which can be normalized by colonization with *Bifidobacterium infantis*, highlighting the critical role of commensal microbes in neuroendocrine development (Ohara and Hsiao, 2025). Subsequent studies further support this bidirectional interaction, showing that gut microbial alterations are associated with gastrointestinal and neuropsychiatric disorders (Person and Keefer, 2021). For example, Hsiao et al. demonstrated that *Bacteroides fragilis* treatment restores intestinal barrier function and improves behavioral abnormalities in a maternal immune activation model (Hsiao et al., 2013), while Sampson et al. showed that gut microbiota from Parkinson’s disease (PD) patients can induce motor deficits in α-synuclein–expressing mice (Sampson et al., 2016).

**Figure 2:**
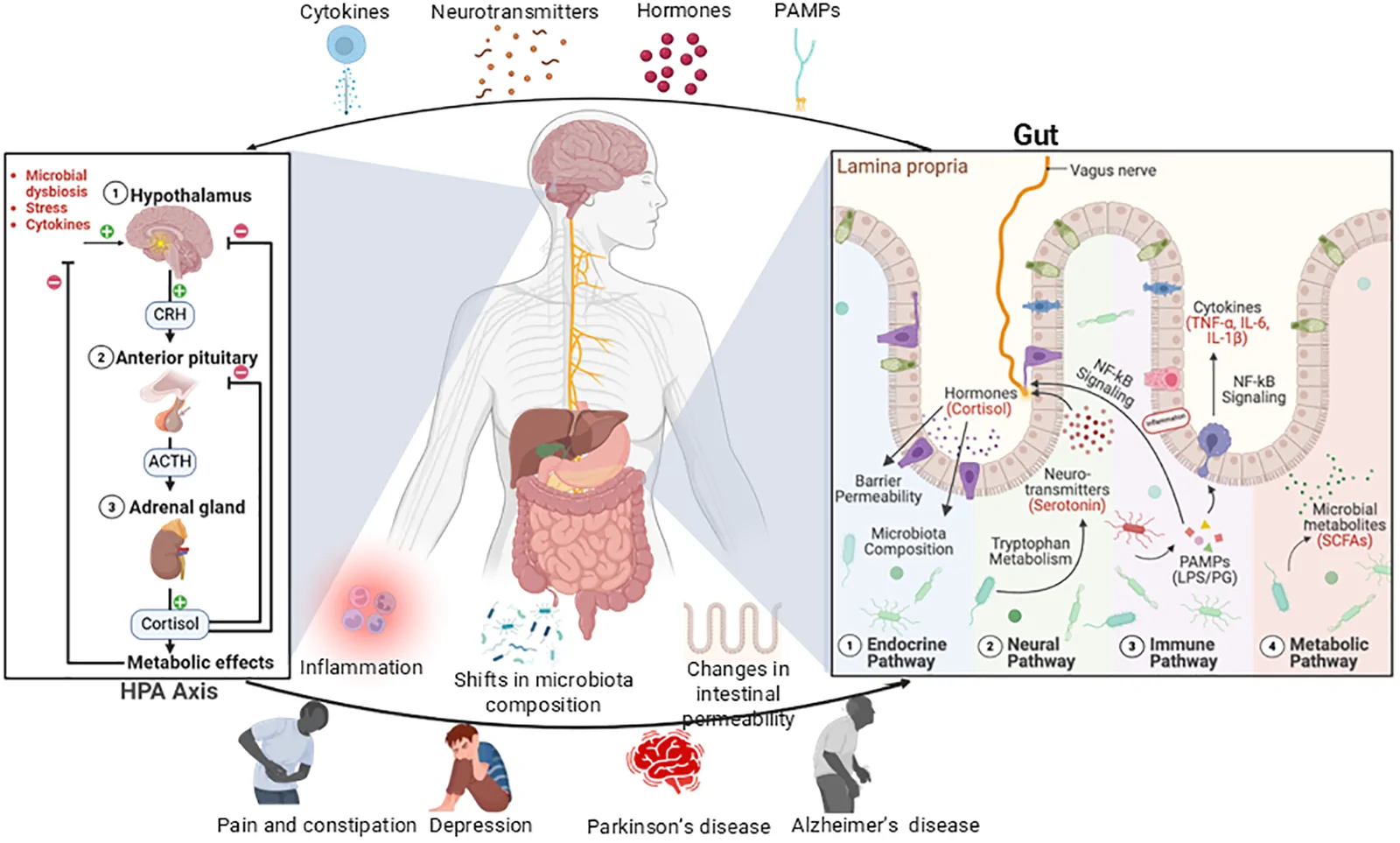
The microbiota-gut-brain axis in health and disease. This schematic illustrates the bidirectional communication network between the gut microbiota, gastrointestinal tract, and central nervous system, collectively known as the microbiota-gut-brain axis. In healthy states, gut microbes produce neuroactive compounds such as short-chain fatty acids (SCFAs), gamma-aminobutyric acid (GABA), serotonin, and dopamine, which influence brain function via neural (vagus nerve), immune, and endocrine pathways. Disruption of microbial balance (dysbiosis) can impair this communication along endocrine, neural, immune, and metabolic pathways, contributing to the development of neuropsychiatric and neurodegenerative disorders such as depression, autism spectrum disorder (ASD), Parkinson's disease, and Alzheimer's disease.

Collectively, these findings demonstrate that the gut–brain axis operates as an integrated bidirectional system in which microbial metabolites shape CNS function, while CNS-derived signals regulate intestinal physiology and microbial ecology. This reciprocal communication influences neurotransmission, synaptic plasticity, immune activation, and epithelial integrity, contributing to the pathogenesis of depression, anxiety, autism spectrum disorder (ASD), (PD, and Alzheimer’s disease (Sudo et al., 2004; Hsiao et al., 2013; Sampson et al., 2016; Person and Keefer, 2021) (**Figure 2**).

## Microbiome in human diseases: IBD, obesity, diabetes, and cancer pathogenesis

The gut microbiome plays a central role in immune and metabolic regulation making it a key contributor to the pathogenesis of multiple human diseases, including BD, obesity, diabetes, and cancer. Across these conditions, microbial alterations are increasingly recognized as key modulators of disease susceptibility, progression, and treatment response.

In IBD, bidirectional interactions between microbial communities and host immune responses significantly influence disease severity and severity (Kumar et al., 2019). Dysbiosis is one of the most consistently reported features of IBD, although its causal role remains unresolved. Characteristic alterations include reduced abundance of Firmicutes and depletion of butyrate-producing taxa such as *F. prausnitzii* and *Roseburia hominis*, alongside enrichment of Proteobacteria, particularly *E. coli* (Machiels et al., 2014; Takahashi et al., 2016; Kumar et al., 2019; Iliev et al., 2025). While these microbial signatures are reproducible across cohorts, their specificity to IBD is limited, and their functional relevance is influenced by substantial inter-individual variability and environmental factors, as also reflected in studies of discordant twins (Willing et al., 2010; Lopez-Siles et al., 2014).

Among candidate organisms, adherent-invasive *E. coli* (AIEC) has been extensively studied due to its enrichment in ileal Crohn’s disease (CD) and its capacity to induce pro-inflammatory responses, including TNFα production (Darfeuille-Michaud and Colombel, 2008; Bucker et al., 2014; Kotlowski, 2016; Ummarino, 2017; Renouf et al., 2018; Zhang et al., 2018). However, its presence in healthy individuals supports a model in which AIEC and similar taxa act as opportunistic pathobionts, with disease potential determined by host susceptibility and ecological context rather than primary causation (Conte et al., 2014; Martinez-Medina and Garcia-Gil, 2014; Zhang et al., 2015; O'Brien et al., 2017). Other proposed microbial contributors, including *Mycobacterium avium* subspecies *paratuberculosis*, *Pseudomonas aeruginosa*, and *Fusobacterium nucleatum*, have shown inconsistent associations in human studies despite supportive experimental evidence (Sasaki et al., 2007; Conte et al., 2014; Martinez-Medina and Garcia-Gil, 2014; Zhang et al., 2015; O'Brien et al., 2017). Collectively, current evidence favors a model in which IBD arises from disrupted microbial community structure and altered host–microbe interactions rather than single infectious agents (Ni et al., 2017).

Mechanistically, a major downstream consequence of dysbiosis is impaired intestinal barrier integrity. Disruption of tight junction architecture leads to increased epithelial permeability, facilitating microbial translocation and persistent mucosal immune activation (Chelakkot et al., 2018; Zuo et al., 2020). Under homeostatic conditions, the mucosal barrier is reinforced by mucus layers and epithelial immune surveillance, both of which are tightly regulated by the gut microbiota (Chelakkot et al., 2018). In dysbiotic states, barrier dysfunction is accompanied by exaggerated immune activation, including dysregulation of autophagy, IL23/IL17 signaling, and Paneth cell function, all of which contribute to chronic intestinal inflammation (Jostins et al., 2012; Mohebali et al., 2023).

In obesity, the microbiome often shifts toward configurations that enhance energy harvest and promote fat storage (Davis, 2016). This is often characterized by an elevated *Firmicutes*-to-*Bacteroidetes* (F/B) ratio, increased circulating LPS levels leading to metabolic endotoxemia, and reduced production of beneficial SCFAs (Magne et al., 2020). These alterations contribute to low-grade systemic inflammation, insulin resistance, and impaired glucose metabolism, thereby increasing susceptibility to T2D (Lu et al., 2024). Mechanistically, LPS activates Toll-like receptor 4 (TLR4)-dependent NF-κB signaling in macrophages and other immune cells, resulting in increased production of pro-inflammatory cytokines, including TNF-α, IL-1β, and IL-6, which disrupt insulin signaling and compromise intestinal barrier integrity (Toshchakov et al., 2002; Arif et al., 2004; Kong et al., 2007; Eun et al., 2014; Marro et al., 2019; Ghosh et al., 2020; Wang et al., 2020) (**Figure 2**). Dysbiosis-associated activation of MyD88-dependent pathways further promotes inflammatory responses, increased intestinal permeability, and bacterial translocation, contributing to metabolic dysfunction and immune activation (Jialal et al., 2012; Jin et al., 2013). Furthermore, enrichment of potentially pro-inflammatory bacterial taxa, including *Bacteroides*, *Ruminococcus*, and *Veillonella*, has been associated with elevated expression of inflammatory cytokines and chemokines, including TNF-α, IL-1β, IL-23A, IL-6, IL-8, CCL3, and CCL4 (de Goffau et al., 2014; Kemppainen et al., 2015; Zhao et al., 2017; Higuchi et al., 2018; Huang et al., 2018; Leiva-Gea et al., 2018; Stewart et al., 2018; Vatanen et al., 2018; Zheng et al., 2018). These alterations are often accompanied by reduced abundances of beneficial SCFA-producing bacteria such as *Bifidobacterium* and *Lactobacillus*, which are important for maintaining intestinal barrier integrity and immune homeostasis (Silva et al., 2020).

In diabetes, increased abundances of *Bacteroides*, *Streptococcus*, and *Lactococcus*, alongside reduced levels of *Bifidobacterium*, *Akkermansia*, and *Ruminococcus*, were reported (Vatanen et al., 2018; Elhag et al., 2021). Comparative analyses between diabetic and non-diabetic individuals further revealed a marked depletion of *Bifidobacterium* and *Lactobacillus*, and enrichment of *Clostridium*, *Bacteroides*, *Ruminococcus*, and *Veillonella* species, associations linked to poor glycemic control and increased intestinal permeability (Zhao et al., 2017; Elhag et al., 2021). Conversely, SCFAs, particularly butyrate, exert potent immunoregulatory effects by promoting M2 macrophage polarization through histone deacetylase (HDAC) inhibition and STAT6 activation, thereby suppressing inflammatory responses (Ji et al., 2016). SCFAs also enhance IL-10 production in T and B cells through mTOR-dependent metabolic and epigenetic pathways, contributing to immune tolerance, maintenance of intestinal barrier integrity, and protection against inflammation-associated β-cell damage (Valladares et al., 2010; Hur and Lee, 2015; Chen et al., 2017; Luo et al., 2017).

In the context of oncology, the gut microbiota influences tumorigenesis through multiple mechanisms, including chronic inflammation, genotoxin production, and immune modulation (Wei et al., 2025). In CRC, the gut microbiota is characterized by enrichment of pathogenic taxa such as *Pseudomonas*, *Helicobacter*, and *Acinetobacter*, alongside depletion of beneficial butyrate-producing bacteria (Zackular et al., 2013). Advanced CRC stages often exhibit elevated levels of *B. massiliensis*, *B. ovatus*, *B. vulgatus*, and *E. coli*, which exacerbate inflammation and promote disease progression (Kostic et al., 2013; Rubinstein et al., 2013; Feng et al., 2015). Among specific microbial drivers, *Fusobacterium nucleatum* is frequently enriched in CRC tissue and has been implicated in multiple tumor-promoting processes, including immune evasion, recruitment of tumor-associated myeloid cells, activation of Wnt/β-catenin signaling via FadA–E-cadherin interaction, induction of autophagy, and promotion of chemoresistance (Yu et al., 2017; Zhu et al., 2024). Similarly, enterotoxigenic *B. fragilis* contributes to carcinogenesis by disrupting epithelial integrity and activating pro-oncogenic signaling pathways such as β-catenin and STAT3, thereby sustaining a chronic inflammatory and proliferative state (Kang et al., 2025; Lei et al., 2025). In parallel, colibactin-producing (pks+) E. coli induces DNA damage and promotes senescence-associated secretory phenotypes that further enhance inflammatory and tumor-promoting signaling (Iftekhar et al., 2021). Collectively, the gut microbiome acts as a key regulator of host metabolism, immunity, and disease progression across metabolic disorders, inflammation, and cancer, primarily through effects on microbial metabolites, immune modulation, and epithelial integrity.

## Microbial biomarkers in predicting and modulating therapeutic response

The gut microbiota plays a pivotal role in shaping drug pharmacokinetics, pharmacodynamics, and host immune responsiveness, thereby influencing both therapeutic efficacy and toxicity (Weersma et al., 2020; Zhao Q. et al., 2023). Direct microbial metabolism can modify drug activity, as illustrated by *Eggerthella lenta*-mediated inactivation of digoxin and bacterial β-glucuronidase–dependent reactivation of irinotecan, which contributes to dose-limiting gastrointestinal toxicity (Cheng et al., 2019). In addition, microbial metabolites—including SCFAs, secondary bile acids, and tryptophan catabolites—regulate hepatic cytochrome P450 enzyme expression, thereby indirectly influencing systemic drug metabolism and clearance (Dempsey and Cui, 2019). Beyond pharmacokinetics, microbiota also modulates host immune priming, cytokine signaling, and inflammatory tone, thereby shaping responses to immunomodulatory and biologic therapies (Akobeng et al., 2020).

In IBD, multiple studies have linked gut microbial composition to differential responses to biologic therapies, particularly anti-TNF agents (Akobeng et al., 2020; Ding et al., 2020; Chopra et al., 2022; Elawad et al., 2022; Kumar et al., 2022; Kumar et al., 2024). Patients with higher microbial diversity and enrichment of beneficial taxa such as *F. prausnitzii*, *Ruminococcus bromii*, *Bifidobacterium* spp., *Clostridium colinum*, and *Eubacterium rectale* tend to exhibit improved therapeutic outcomes, whereas reduced diversity and enrichment of inflammatory taxa are associated with non-response (Kolho et al., 2015; Singh et al., 2016; Lee et al., 2021). Enrichment of butyrate-producing bacteria such as *Roseburia inulinivorans* and *Burkholderiales*, together with elevated branched-chain amino acid levels, has also been linked to positive clinical outcomes in patients treated with Vedolizumab (Ananthakrishnan et al., 2017). Conversely, specific microbial configurations enriched in SCFA-producing families such as *Lachnospiraceae* and *Ruminococcaceae* have also been reported in non-responders, highlighting that microbial function, strain-level variation, and metabolic output may be more informative than taxonomy alone Yilmaz et al., 2019).

Mechanistically, beneficial microbial communities enhance therapeutic responsiveness through the production of SCFAs, particularly butyrate, which promotes regulatory T-cell differentiation, suppresses pro-inflammatory cytokine production, strengthens epithelial barrier integrity, and supports mucosal healing (Lavelle and Sokol, 2020). In contrast, dysbiosis contributes to impaired barrier function, increased permeability, and heightened systemic inflammation, all of which are associated with reduced response to anti-TNF and anti-integrin therapies (Frin et al., 2017). Complementary host-derived inflammatory biomarkers, including IL-6, TNF-α, IL-1β, IL-8, IL-10, IFN-γ, and soluble TNF receptor 2 (sTNFR2), are frequently elevated in non-responders (Baird et al., 2016; Billiet et al., 2017; Bertani et al., 2020; Bertani et al., 2020). The compositional balance between dominant microbial genera, particularly *Prevotella* and *Bacteroides*, has also been explored in relation to treatment efficacy (Larsen, 2017; Bazin et al., 2018). A higher *Prevotella/Bacteroides* ratio has been correlated with improved clinical response to biologic therapy (Franzin et al., 2021). Microbial imbalance further contributes to impaired intestinal barrier function, increased permeability, and immune activation, collectively driving chronic intestinal inflammation, thereby reinforcing the interaction between systemic inflammation and microbial imbalance in determining therapeutic outcomes (Elhag et al., 2022).

Beyond IBD, the gut microbiome plays a critical role in modulating responses to immune checkpoint inhibitors (ICIs), including anti-CTLA-4 and anti-PD-1/PD-L1 therapies (Iida et al., 2013; Zhao L-Y. et al., 2023). Preclinical studies demonstrate that antibiotic treatment or germ-free conditions impair anti-tumor immunity and significantly reduce ICI efficacy, largely due to defective myeloid cell activation and reduced pro-inflammatory cytokine production (Iida et al., 2013; Sivan et al., 2015; Vétizou et al., 2015). Restoration of microbial communities with specific taxa such as *B. fragilis*, *Burkholderia cepacia*, or *B. thetaiotaomicron*, rescues dendritic cell maturation, enhances Th1 polarization, and restores CD8^+^ T-cell–mediated anti-tumor responses (Vétizou et al., 2015). Notably, some of these microbes also mitigate therapy-induced colitis, suggesting dual immunotherapeutic and protective roles (Dubin et al., 2016).

**Figure 3:**
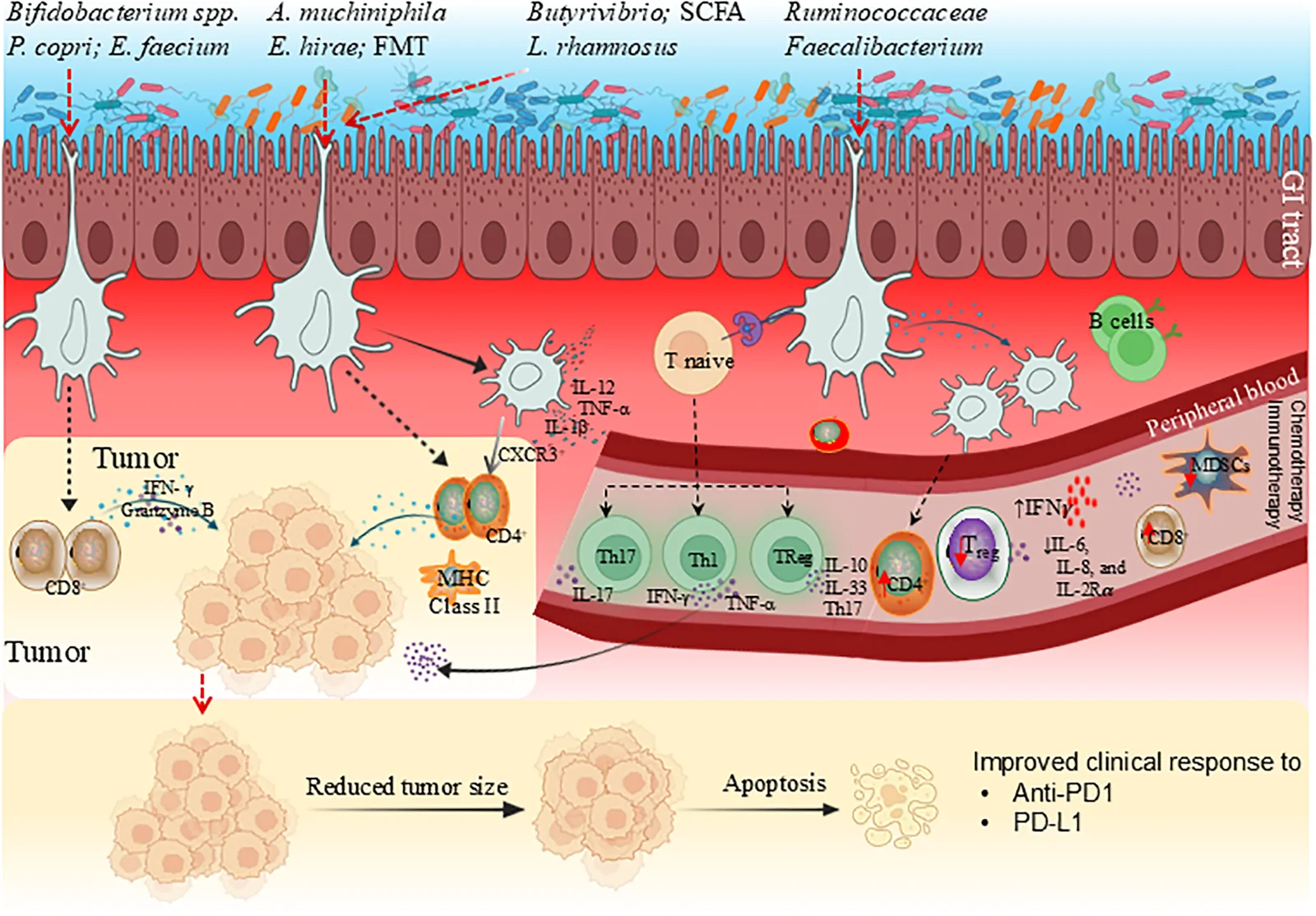
Potential influences of gut microbiota on the efficacy of anticancer therapy. The presence of specific microbial species correlate with increased levels of effector CD4^+^ and CD8^+^ T cells, enhanced secretion of cytokines such as IL-10 and IL-33, and elevated production of chemokines that recruit T cells. Baseline gut microbiota composition ennched with *Akkermansia muciniphila*, *Bifidobacterium* spp., *Butyrivibrio*, *Faecalibacterium* spp., and members of the *Ruminococcaceae* family has been associated with improved responses to immunotherapy in melanoma patients. These beneficial microbes modulate various immune cell populations, including dendritic cells (DCS), T helper 1 (TH1), T helper 17 (TH17), regulatory T (Treg) cells, CD8^+^ cytotoxic T cells, and B cells, promoting the production of inflammatory cytokines such as IFNy and TNF in the blood to promote the efficacy of anti-tumor therapy.

Clinical studies further confirm that responder-associated microbiota are enriched in patients with improved outcomes across melanoma, non-small cell lung cancer (NSCLC), and renal cell carcinoma (RCC) composition (Gopalakrishnan et al., 2018; Matson et al., 2018; Routy et al., 2018). Key taxa include *A. muciniphila*, *F. prausnitzii*, *Bifidobacterium longum*, *Ruminococcaceae*, and *Enterococcus hirae* which collectively enhance dendritic cell function, antigen presentation, and CD8^+^ T-cell activation (Gopalakrishnan et al., 2018; Matson et al., 2018; Routy et al., 2018). Mechanistically, these microbes promote a more immunogenic tumor microenvironment by increasing IFN-γ production (Gopalakrishnan et al., 2018), enhancing cytotoxic CD8^+^ T-cell infiltration, and modulating regulatory immune populations such as increased IFN-γ production, and enhanced tumor immune infiltration (Shi et al., 2025; Xie et al., 2025). Specifically, *Bifidobacterium* spp. improves cross-presentation and MHC-II expression (Sivan et al., 2015), while *A. muciniphila* supports mucosal barrier integrity and immune signaling, and *F. prausnitzii* contributes anti-inflammatory effects via butyrate production (Derosa et al., 2018; Routy et al., 2018; Shi et al., 2025; Chen et al., 2026). Collectively, these microbiota-driven effects induce a more inflamed and immunotherapy-responsive tumor microenvironment (**Figure 3**) while reducing pathways associated with immune suppression and treatment resistance (Lei et al., 2025), ultimately contributing to improved clinical outcomes (**Table 3**).

Conversely, prior exposure to antibiotics has been linked to impaired therapeutic response (Derosa et al., 2018; Routy et al., 2018), likely due to disruption of beneficial microbial communities. Beyond their role in modulating therapeutic efficacy, microbiota also serves as potential biomarkers for cancer detection, progression, and treatment stratification. For instance, *Helicobacter pylori* is implicated in gastric cancer (IARC Working Group on the Evaluation of Carcinogenic Risks to Humans, 1994), while *F. nucleatum* is associated with colorectal cancer and poor prognosis (Kostic et al., 2013; Rubinstein et al., 2013; Vétizou et al., 2015). Similarly, alterations in oral and gut microbiota profiles are being explored as predictive markers for pancreatic, liver, and other cancers (Attebury and Daley, 2023; Chen et al., 2023; Zhou et al., 2024).

Emerging therapeutic strategies aim to harness microbiomes to enhance cancer treatment outcomes. Approaches include fecal microbiota transplantation (FMT) from responders (Fessler et al., 2024), administration of probiotics or next-generation bacterial consortia such as MET-4, VE800, and EDP1503 [(Spreafico et al., 2023; Lyon De Ana et al., 2024), NCT03775850], and dietary modulation (Golonko et al., 2024). These interventions have shown promise in improving ICI responsiveness, reversing resistance, and mitigating immune-related adverse events. Several ongoing clinical trials continue to explore these strategies as adjuncts to conventional cancer immunotherapy (NCT04988841; NCT04636775; NCT03637803), with the goal of improving response rates, overcoming resistance, and reducing immune-related adverse events.

## Challenges in clinical translation of the human microbiome

Although microbiome research has shown substantial translational potential, several practical barriers continue to limit its integration into routine clinical practice. Variability in sample collection, sequencing technologies, bioinformatic pipelines, and analytical approaches, together with differences in sequencing depth, contamination, incomplete reference databases, and methodological inconsistencies, continue to hinder reproducibility and complicate cross-study comparisons and data interpretation (Knight et al., 2018; Schloss, 2018; Chorlton, 2024; Treichel et al., 2026). In addition, factors such as dietary habits, antibiotic exposure, lifestyle, age, geography, host genetics environmental exposures, medications, and baseline microbiota composition contribute to considerable inter-individual variability in microbiome composition and to inconsistent therapeutic outcomes (Qin et al., 2022; Van Hul et al., 2024; Govender and Ghai, 2025; Qu et al., 2025). These challenges are compounded by the difficulty of defining stable reference states, as microbial profiles vary dynamically across individuals and over time, making the concepts of “eubiosis” and “dysbiosis” difficult to universally define.

Clinical translation is also limited by safety, regulatory, and implementation challenges. Early microbiome-based interventions, particularly FMT, have raised concerns regarding pathogen transmission, unintended immune effects, and ecological disruption of the native microbiota (DeFilipp et al., 2019). Additional barriers include donor selection, manufacturing standardization, ethical considerations, patient acceptance, and high implementation costs (Kelly et al., 2015; O’Toole et al., 2017). Although regulatory frameworks from agencies such as the FDA and EMA are beginning to emerge (Rodriguez et al., 2025), further progress is required to establish standardized methodologies, robust validation strategies, large longitudinal datasets, and advanced analytical approaches, including AI-driven tools (Metwaly et al., 2025; Ponce de Leon-Derecho and Dable-Tupas, 2025). Collectively, these challenges highlight that while the microbiome represents a highly promising frontier in precision medicine, significant methodological and translational hurdles remain to be addressed.

### Future perspective

Over the past two decades, advances in microbiome science have profoundly reshaped our understanding of host–microbe interactions and their impact on human health and disease. The next generation of studies now aims to elucidate the functional roles of microbial genes, transcripts, proteins, and metabolites within a systems biology framework (Ma et al., 2024). This shift is essential not only for a comprehensive understanding of microbiome contributions to health and disease, but also for identifying robust, clinically actionable microbial biomarkers as well as therapeutics (Charbonneau et al., 2020; Passaro et al., 2024). Increasing evidence suggests that microbial metabolites, including SCFAs, secondary bile acids, and tryptophan-derived compounds, act as key mediators of host–microbe communication by regulating epithelial barrier integrity, immune cell differentiation, cytokine signaling, and metabolic homeostasis (Lei et al., 2025). Mechanistically, SCFAs such as butyrate promote regulatory T-cell differentiation, suppress pro-inflammatory signaling through histone deacetylase inhibition, enhance intestinal barrier function, and support mucosal healing (Sankarganesh et al., 2025; Chen H. et al., 2026). Similarly, microbial bile acid metabolism influences FXR and TGR5 signaling pathways (Song et al., 2025), whereas tryptophan metabolites modulate AhR-dependent immune responses and IL-22-mediated epithelial repair (Seo and Kwon, 2023), highlighting the diverse mechanisms through which the microbiome shapes host physiology and disease outcomes. Recent studies have further demonstrated the therapeutic relevance of these mechanisms in cancer immunotherapy. Specific microbial taxa, including *A. muciniphila*, *F. prausnitzii*, *B. longum*, and *E. hirae*, have been associated with improved responses to ICIs through enhanced dendritic cell maturation, antigen presentation, CD8^+^ T-cell activation, and IFN-γ production, ultimately promoting anti-tumor immunity and favorable remodeling of the tumor microenvironment (Gopalakrishnan et al., 2018; Matson et al., 2018; Routy et al., 2018). In contrast, germ-free or antibiotic-treated models exhibit diminished responsiveness to ICIs, underscoring the necessity of a functional and intact microbiome for optimal therapeutic efficacy (Iida et al., 2013; Sivan et al., 2015; Vétizou et al., 2015).

Despite these promising insights, microbiome science remains far from full integration into clinical practice. Establishing causal relationships between the gut microbiome and disease continues to be challenging due to its immense complexity, inter-individual variability, and the influence of host genetics, diet, lifestyle, medication use, and environmental factors (Whelan et al., 2024; Porcari et al., 2025; Turjeman et al., 2025). Furthermore, microbiome profiles often vary across populations and geographical regions, limiting the reproducibility and generalizability of findings. Many reported microbial biomarkers lack validation in large, independent cohorts, reducing their immediate clinical applicability (Porcari et al., 2025; Turjeman et al., 2025). Additional barriers include mechanistic uncertainty, difficulties in achieving stable microbial engraftment following microbiome-based interventions (Herman et al., 2025), and challenges in manufacturing, standardizing, and regulating live biotherapeutic products (Kelly et al., 2015; O’Toole et al., 2017; Pan et al., 2025). Safety concerns, including the potential transfer of pathogenic organisms (DeFilipp et al., 2019), antimicrobial resistance genes, and unintended immune effects, also require careful consideration before widespread clinical implementation. Moreover, the absence of harmonized regulatory frameworks and standardized protocols for sample collection, sequencing, data analysis, and reporting complicates comparisons across studies and slows clinical adoption.

To address these limitations, future studies should prioritize longitudinal, multicenter investigations with diverse populations and standardized methodologies. Adoption of reporting frameworks such as the STORMS checklist, combined with rigorous clinical trial design and integration of metagenomics, metatranscriptomics, metabolomics, proteomics, and host immune profiling, will improve reproducibility and facilitate mechanistic discovery (van der Loos et al., 2019; Lloyd-Price et al., 2019; Mirzayi et al., 2021; The Integrative Human Microbiome Project, 2019). In parallel, developing educational programs, workshops, and collaborative networks that equip clinicians with microbiome expertise will facilitate the seamless integration of microbiome science into patient care (Porcari et al., 2025; Turjeman et al., 2025).

Looking ahead, however, the field is clearly shifting from descriptive profiling toward mechanistic and translational microbiome research. Integrative multi-omics technologies and systems biology frameworks will be essential for uncovering functional microbial signatures and guiding the development of targeted microbiome-based interventions (Zitvogel et al., 2018; Lloyd-Price et al., 2019; Piccinno et al., 2025). These approaches will facilitate the development of next-generation microbiome-based interventions, including precision probiotics, engineered live biotherapeutics, microbial metabolite-based therapeutics, targeted bacteriophage therapies, and personalized fecal microbiota transplantation strategies. With an increasing number of clinical trials evaluating microbiome-targeted interventions across inflammatory, metabolic, neurological, and oncological diseases, there is growing optimism that microbiome-informed diagnostics, prognostics, and therapeutics will soon become a cornerstone of precision medicine (Spreafico et al., 2023; Fessler et al., 2024; Lyon De Ana et al., 2024). Taken together, a deeper mechanistic understanding of the microbiome’s role in health and disease, combined with technological and translational advances, will pave the way for personalized, microbiota-targeted therapies that can enhance treatment efficacy, reduce toxicity, and improve long-term outcomes across diverse clinical contexts.
