# Supplementary material for: Decoding the power of the microbiome in human health
Source: Front Cell Infect Microbiol. 2026 Aug 21;16:1877371. doi: 10.3389/fcimb.2026.1877371 (PMC13542889; doi:10.3389/fcimb.2026.1877371)
Supplement: Supplementary file 1 [file DataSheet1.docx]

Supplementary table S1: Antibiotic Classes, Producers, Clinical Use, and Natural Habitat.

| Antibiotic Class | Antibiotic | Producing Bacterium | Clinical Use | Natural Habitat | Ref |
| --- | --- | --- | --- | --- | --- |
| β-lactam (Penicillin) | Penicillin | *Penicillium notatum,  Penicillium chrysogenum* | Gram-positive infections; syphilis, streptococcal diseases | Soil, decaying fruit/organic matter | (1) |
|  |  |  |  |  |  |
| Aminoglycoside | Streptomycin | *Streptomyces griseus* | Tuberculosis, plague, Gram-negative infections | Soil (decaying vegetation) | (2) |
| Aminoglycoside | Gentamicin | *Micromonospora purpurea* | Severe Gram-negative infections | Marine and freshwater sediments | (3) |
| Macrolide | Erythromycin | *Streptomyces erythraeus* | Respiratory infections, mycoplasma pneumonia | Soil (alkaline forest soils) | (4) |
| Tetracycline | Tetracycline | *Streptomyces aureofaciens* | Acne, respiratory, urinary infections | Agricultural soils | (5) |
| Amphenicol | Chloramphenicol | *Streptomyces venezuelae* | Broad-spectrum; reserved due to toxicity | Tropical and temperate soil | (6) |
| Macrolide-like | Avermectins | *Streptomyces avermitilis* | Antiparasitic (e.g., ivermectin) | Soil (Japan) | (7) |
| β-lactamase Inhibitor | Clavulanic acid | *Streptomyces clavuligerus* | β-lactam combinations (e.g., Augmentin) | Soil (Italy) | (8) |
| Glycopeptide | Vancomycin | *Streptomyces orientalis* | MRSA, severe Gram-positive infections | Soil (Borneo rainforest) | (9) |
| Rifamycin | Rifampicin | *Streptomyces rifamycinica* | Tuberculosis, leprosy | Soil (French Riviera) | (10) |
| Polypeptide | Bacitracin | *Bacillus subtilis* | Topical for skin infections | Soil, plant surfaces | (11), |
| Polypeptide | Gramicidin | *Bacillus brevis* | Topical for Gram-positive infections | Soil, compost | (12) |
| Polypeptide | Polymyxin B, E | *Bacillus polymyxa* | MDR Gram-negative infections | Soil, rhizosphere | (13) |
| β-lactam (Penicillin-like) | Nocardicin | *Nocardia spp.* | Experimental β-lactam antibiotic | Soil, decaying vegetation | (14) |
| Polypeptide (Cytotoxic) | Actinomycin D | *Actinomyces spp.* | Chemotherapy (not typical antibiotic use) | Soil, oral cavity | (15) |

References

1.	Shaheen M, Li J, Ross Avena C, Vederas John C, Jensen Susan E. 2011. Paenibacillus polymyxa PKB1 Produces Variants of Polymyxin B-Type Antibiotics. Chemistry & Biology 18:1640-1648.

2.	Schatz A, Bugle E, Waksman SA. 1944. Streptomycin, a Substance Exhibiting Antibiotic Activity Against Gram-Positive and Gram-Negative Bacteria.∗†. Proceedings of the Society for Experimental Biology and Medicine 55:66-69.

3.	Gonzalez R, Islas L, Obregon AM, Escalante L, Sanchez S. 1995. Gentamicin formation in Micromonospora purpurea: stimulatory effect of ammonium. J Antibiot (Tokyo) 48:479-83.

4.	Oliynyk M, Samborskyy M, Lester JB, Mironenko T, Scott N, Dickens S, Haydock SF, Leadlay PF. 2007. Complete genome sequence of the erythromycin-producing bacterium Saccharopolyspora erythraea NRRL23338. Nature Biotechnology 25:447-453.

5.	Duggar BM. 1948. Aureomycin; a product of the continuing search for new antibiotics. Ann N Y Acad Sci 51:177-81.

6.	Ehrlich J, Bartz QR, Smith RM, Joslyn DA, Burkholder PR. 1947. Chloromycetin, a New Antibiotic From a Soil Actinomycete. Science 106:417.

7.	Guo J, Ma R, Su B, Li Y, Zhang J, Fang J. 2016. Raising the avermectins production in Streptomyces avermitilis by utilizing nanosecond pulsed electric fields (nsPEFs). Scientific Reports 6:25949.

8.	Reading C, Cole M. 1977. Clavulanic acid: a beta-lactamase-inhiting beta-lactam from Streptomyces clavuligerus. Antimicrob Agents Chemother 11:852-7.

9.	McCormick MH, McGuire JM, Pittenger GE, Pittenger RC, Stark WM. 1955. Vancomycin, a new antibiotic. I. Chemical and biologic properties. Antibiot Annu 3:606-11.

10.	Sensi P. 1983. History of the development of rifampin. Rev Infect Dis 5 Suppl 3:S402-6.

11.	Rietkötter E, Hoyer D, Mascher T. 2008. Bacitracin sensing in Bacillus subtilis. Mol Microbiol 68:768-85.

12.	Katz E, Demain AL. 1977. The peptide antibiotics of Bacillus: chemistry, biogenesis, and possible functions. Bacteriol Rev 41:449-74.

13.	Shaheen M, Li J, Ross AC, Vederas JC, Jensen SE. 2011. Paenibacillus polymyxa PKB1 produces variants of polymyxin B-type antibiotics. Chem Biol 18:1640-8.

14.	Aoki H, Sakai H, Kohsaka M, Konomi T, Hosoda J. 1976. Nocardicin A, a new monocyclic beta-lactam antibiotic. I. Discovery, isolation and characterization. J Antibiot (Tokyo) 29:492-500.

15.	Waksman SA, Woodruff HB. 1940. Bacteriostatic and Bactericidal Substances Produced by a Soil Actinomyces.∗. Proceedings of the Society for Experimental Biology and Medicine 45:609-614.
